# Editorial: From batch-size 1 to serial production: adaptive robots for scalable and flexible production systems

**DOI:** 10.3389/frobt.2023.1201488

**Published:** 2023-04-26

**Authors:** Mohamad Bdiwi, Steffen Ihlenfeldt

**Affiliations:** Fraunhofer Institute for Machine Tools and Forming Technology (FHG), Chemnitz, Germany

**Keywords:** agile manufacturing and industry 4.0, from simulation to real robot applications safety and virtual commissioning, flexible robotics for handiwork, AI and perception, robot control

The first manuscript with the title “Halim et al.” proposes a no-code approach to programming industrial robots. The proposed method relies on a finite state machine with three layers of natural interactions based on hand gestures, finger gestures, and voice recognition. The results obtained from the experiments indicate the capability of this novel approach for real-world deployment in an industrial context. In a similar vein, however, on the controller level, the third manuscript “Wiese et al.” presents a skill-based approach as an abstract template class methodically for modularization of the assets in the control and parameterizable skills. An orchestration system is used to call the skills with the corresponding parameter set and combine them into automated process sequences. This approach provides a more flexible control, as operators can independently adapt and expand the automated process sequence without modifying the controller code.

In the second manuscript, “Bdiwi et al.” the authors propose a dynamic safety-related finite-state machine for safe transitions between various collaborative operation modes dynamically and adequately. In addition to that, the collaborative operation modes are grouped in different clusters and categorized at various levels systematically. The proposed approach is integrated into a new dynamic risk assessment tool as a promising solution toward a new safety horizon in line with Industry 4.0.

To enable the production of large components using industrial robots, the fourth manuscript “Schnellhardt et al.” presents a novel approach to segmented manufacturing. The proposed segmentation strategy divides the part into segments whose structural design is adapted to the capabilities of the field components available on the shop floor. The process planning step of each segment is automated by utilizing the similarity of the segments and the self-description of the corresponding field component. The result is a transformation of a batch size one production into an automated quasi-serial production of the segments. Moreover, the fifth manuscript with the title “Wabner et al.” proposes technological cooperation of industrial robots and machine tools to improve flexibility and efficiency in parts production. The approach results in a novel type of collaborative manufacturing equipment for matrix production that will improve the versatility, efficiency, and profitability of production. By enhancing machine tools with additional manufacturing technologies, a robot can beneficially support workpiece machining.

Regarding shop floor management, modern manufacturing objectives such as automation, mass customization, self-organization, and smart factories require intelligent control approaches. The sixth manuscript “Bahrpeyma and Reichelt” presents a review (MARL) as an effective approach to handling uncertainties due to the dynamic nature of the environment. It has been demonstrated how different aspects of smart factories match the objectives and capabilities of MARL and suggested a mapping from smart factory features to the equivalent concepts in MARL, indicating how MARL provides an appropriate solution to provide almost all the required features in the smart factory at once. In terms of robot path planning, most motion planners generate low-level control inputs, leading to geometric and temporal deviations between the executed and planned motions of the robot. To solve this challenge, the work in the seventh manuscript “Hou et al.” proposed a new approach using neural networks. This approach uses realistic collision-free trajectories to simultaneously learn high-level motion commands and robot dynamics, generating trajectories that can be executed directly by the robot control system. The proposed approach has significantly reduced geometric and temporal deviation between the executed and planned motions and generates new collision-free trajectories up to ten times faster than benchmark motion planners.

The last two manuscripts discuss innovative approaches to improve industrial human-robot collaboration using intelligent sensor systems with a focus on safety and efficiency. The eighth manuscript with the title “Krusche et al.” proposes a novel approach for automatic annotation of human actions in 3D point clouds using various DNN classifiers, an intuitive GUI, and a methodology for automatic sequence matching. The proposed framework was evaluated in an industrial use case and was shown to accelerate the annotation process by 5.2 times through automation. This approach can save time and resources while improving human-robot collaboration by recognizing, analyzing, and modeling human actions. The last manuscript with the title “Rashid et al.” presents flexible and efficient local and global sensing using cameras and LiDAR. The proposed methodology incorporates a local 3D sensor on the robot body and formulates occlusion due to the robot body, which ensures minimum occlusion in the robot workspace. The resulting system enables high robot velocities while providing flexibility and safety with heavy-duty industrial robots. The proposed approach aims to have a minimum scalable sensor concept and adjust it according to the process requirements.

